# Unleashing the potential of extracellular vesicles for ulcerative colitis and Crohn's disease therapy

**DOI:** 10.1016/j.bioactmat.2024.11.004

**Published:** 2024-11-11

**Authors:** George Chigozie Njoku, Cathal Patrick Forkan, Fumie Mitani Soltysik, Peter Lindberg Nejsum, Flemming Pociot, Reza Yarani

**Affiliations:** a Translational Type 1 Diabetes Research, Department of Clinical and Translational Research, Steno Diabetes Center Copenhagen, Herlev, Denmark; b Department of Medical Biotechnology, University of Naples Federico II, Naples, Italy; c Department of Cellular and Integrative Physiology, University of Nebraska Medical Center, USA; d Department of Pharmacy, Université Grenoble Alpes, France; e Department of Clinical Medicine, Aarhus University, Aarhus, Denmark; f Department of Infectious Diseases, Aarhus University Hospital, Aarhus, Denmark

## Abstract

• Comprehensive review of MSC-derived EVs as therapeutic agents for IBD.
• MSC-EVs modulate immune responses and promote tissue repair in CD and UC.
• EVs enhance therapeutic effects and reduce toxicity as drug delivery systems in preclinical colitis models.
• Current clinical studies assess the safety and efficacy of MSC-derived EVs for IBD treatment.
• Key challenges scaling production, standardizing isolation addressing EV heterogeneity, and optimizing delivery.

## Graphical abstract

**Figure:**
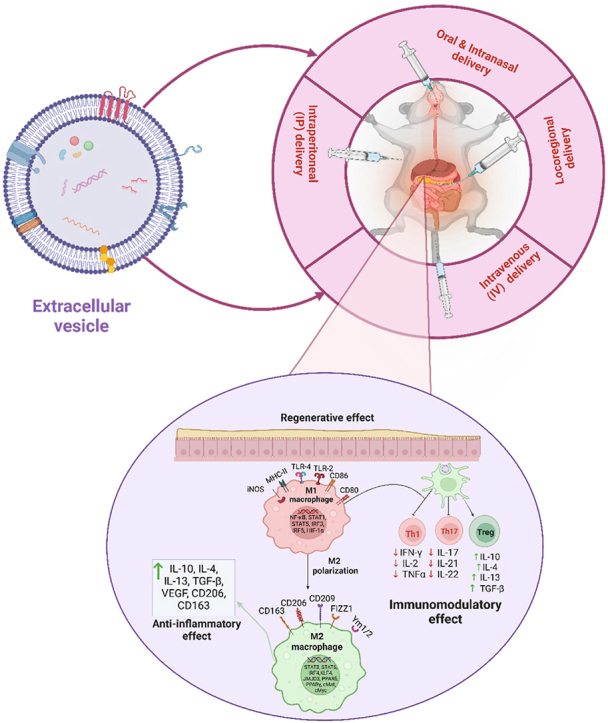

## 1 Introduction

The quality of life for millions of people around the world is severely diminished by the crippling symptoms of inflammatory bowel diseases (IBD), such as Ulcerative Colitis (UC) and Crohn's Disease (CD), which are relapsing inflammatory disorders of the gastrointestinal tract (GIT) [1]. IBD may have significant effects, including strictures, fistulas, and an increased risk of colon cancer [2]. CD and UC have distinct pathology and immunological backgrounds but overlap many clinical characteristics. While UC commonly affects the colon and rectum, resulting in a continuous zone of inflammation, CD may affect any portion of the GIT from mouth to anus, often in a discontinuous pattern [3,4]. Environmental variables, abnormalities in gut microbiota, immune system dysregulation, and genetic predispositions contribute to the pathophysiology of IBD [5]. Adverse side effects, suboptimal efficacy, and the potential for non-response or diminished response over time constrain the existing therapy landscape. This is despite significant advances in understanding these diseases. The therapeutic options include immunosuppressants [6], biological agents [7], and corticosteroids [8]. After ten years of diagnosis, surgical treatments are still needed in 10–20 % of UC patients and 50 % of CD individuals [9]. This emphasizes the paramount need for developing novel therapy approaches to successfully address the underlying inflammatory pathways and maintain remission over the long term [10].

The immunomodulatory and tissue-healing properties of mesenchymal stem cells (MSCs) have garnered significant attention recently, making cell-based treatments a viable therapy option for IBD [11,12]. MSCs emit diverse bioactive substances, such as growth factors and anti-inflammatory cytokines, that can affect immune responses and accelerate tissue regeneration [13]. Nevertheless, the therapeutic deployment of MSCs is impeded by heterogeneity in effectiveness, potential tumorigenicity, manufacturing scalability, and regulatory obstacles [14]. There has been a rapid increase in utilizing extracellular vesicles (EVs) produced by MSCs to resolve these concerns. EVs are membrane-bound, nanoscale particles that are spontaneously released by cells. They include various proteins, lipids, RNAs, and DNAs that impact cellular processes via intercellular communication [15]. EVs are active in various pathological and physiological cellular processes, including cell proliferation, differentiation, migration, survival, immune response, angiogenesis, and drug resistance [16]. MSC-derived EVs (MSC-EVs) mimic many of the positive effects of MSCs and provide significant benefits over whole-cell treatments [17].

In several *in vitro* and *in vivo* models of IBD, MSC-EVs have shown promise in reducing immune responses, accelerating tissue healing, and restoring intestinal homeostasis [18,19]. A study by Wei et al. showed that human umbilical cord mesenchymal stem cell-derived EVs (hucMSC-EVs), through miR-129–5p targeting ACSL4, inhibit ferroptosis and lipid peroxidation in intestinal epithelial cells, thereby alleviating IBD and promoting tissue repair [20]. Lee et al. also demonstrated that EVs derived from adipose-tissue mesenchymal stem cells (ASC-EVs) can relieve symptoms of IBD in a mouse model. They showed that intravenous administration of ASC-EVs was more effective than intraperitoneal delivery, leading to reduced weight loss, improved histological outcomes, and lower disease activity index (DAI). Additionally, a genetic combination of ASC-EVs with an anti-IL-12 antibody significantly enhanced therapeutic outcomes, underscoring the potential of ASC-EVs in IBD treatment and highlighting the importance of optimizing administration routes and dosage for maximal therapeutic benefit [21].

Given their unique capabilities, EVs present a promising therapeutic approach for managing IBD. EV-based therapies offer targeted, cell-free treatments that may reduce the side effects of conventional methods. They provide opportunities for more effective, personalized therapies that could improve patient outcomes while lowering the trial-and-error approach often seen in IBD management [22]. Furthermore, EVs can serve as advanced drug delivery systems, encapsulating medications and delivering them directly to inflamed tissues, which helps minimize systemic exposure and related side effects. Their lower immunogenicity also makes them suitable for repeated use, which is essential for chronic conditions like IBD [23]. However, the potential risks of EV therapy must be considered. While EVs are generally less immunogenic than whole cells, there is still a chance of triggering unwanted immune responses or interactions with the host's immune system. High doses or long-term use of EVs could affect hemostasis, leading to concerns about the risk of thrombosis [17]. Additionally, variability in EV production—such as differences in source cells and isolation methods—can impact their consistency and therapeutic effectiveness, posing challenges for standardization and large-scale manufacturing [24]. Regulatory and safety issues must be addressed to ensure EV-based therapies are safe and effective for clinical use.

This review explores recent progress in EV-based therapies for IBD, focusing on their ability to modulate immune responses, promote tissue repair, and restore intestinal balance. We also examine their potential as drug delivery systems and discuss the challenges and future directions for translating these therapies into clinical practice.

## 2 Extracellular vesicles (EVs)

### 2.1 EV properties

**Fig. 1:**
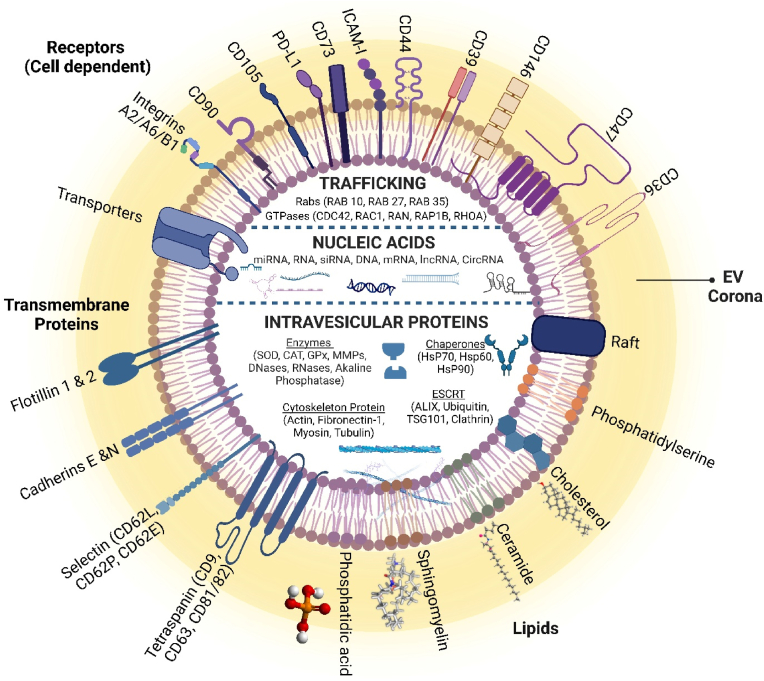
Composition of EVs. EVs exhibit a complex composition of proteins, lipids, and nucleic acids, essential for intercellular communication and disease regulation. Key components include Tetraspanins (CD9, CD63, CD81), MVB-related proteins (TSG101, ALIX, Rab 11, 27, 35), chaperones (HSP90, HSP60, HSP70), integrins, ICAM-1, and specific therapeutic markers (CD73, CD90, CD39, CD146, CD36, CD105, CD44). EVs also transport various RNA species (mRNA, miRNA, lncRNA, piRNA, CircRNA) and DNA forms (CircDNA, mtDNA, ssDNA, dsDNA). The lipid composition includes sphingomyelin, gangliosides, desaturated lipids, cholesterol, and phosphatidylserine, acting as signaling molecules crucial for cell signaling pathways.

EVs are membranous structures crucial for cell-to-cell communication and serve as delivery vehicles for signal molecules. They comprise a phospholipid bilayer enclosing nucleic acids, proteins, lipids, and other cellular metabolites [25]. The composition of EVs varies with the parent cell type properties, EV biogenesis, and size. EVs encompass proteins essential for homeostasis regulation and communication. They also carry cell-dependent markers, including MHC class II, tetraspanins (CD9, CD63, CD81, CD82, flotillin), multivesicular bodies (MVBs) related proteins (TSG101, ALIX, Rab 11, 27 and 35), heat shock proteins (HSP90, HSP70), growth factors, cytokines (TNF-α, VEGF, EGF, TGF-β), adhesion molecules (integrins, ICAM-1) and many more bioactive molecules [26]. EVs also carry proteins important in regulating biological processes involved in antigen presentation, signaling (GTPase HRas, Ras-related protein, Src, RhoA), cytoskeletal components, metabolic enzymes, death receptors (FasL), and iron transport proteins (Fig. 1).

EVs derived from cells, including stem cells, progenitor cells, endothelial cells, and stromal cells, offer significant advantages over traditional cell therapies, such as lower immunogenicity, reduced risk of tumor formation, and more accessible storage and handling requirements [[27], [28], [29]]. EVs do not carry a complete genome or have the capacity to proliferate, which minimizes the risk of tumor formation that can be associated with stem cell therapies [30,31]. These vesicles also show promising effects in tissue repair and regeneration and play a role in modulating the immune system by reducing auto-reactive T cells and increasing regulatory T cells (Tregs) [29]. As promising therapeutic candidates in regenerative medicine, EVs are being explored for treating conditions like type 1 diabetes [32,33], rheumatoid arthritis [34], cancer [35], and neurological diseases [36]. For instance, EVs derived from MSCs exhibit potent regenerative capabilities, while those from endothelial progenitor cells are known for their proangiogenic properties [37]. Engineering EVs to enhance their therapeutic potential and using them for targeted drug delivery could offer an innovative approach in biological nanotherapeutics for precision medicine [38].

### 2.2 EV biogenesis

EVs have seen significant advancements, identifying various subtypes with unique characteristics. Among these subtypes are exosomes (Exo), microvesicles (MVs), apoptotic bodies (ABs), and more recently discovered entities such as microsomes, exospheres, large oncosomes (LOs), autophagosomes, ectosomes [39,40], and non-vesicular extracellular particles (NVEPs) like supermeres and exomers [41,42], as illustrated in Table 1. To ensure consistency and accuracy in nomenclature, this review adheres to the guidelines set by the International Society for Extracellular Vesicles (ISEV) as outlined in their consensus paper, Minimal Information for Studies of Extracellular Vesicles (MISEV2023) [43]. For this review, the term “EVs” will refer to all forms of EVs unless identified explicitly as exosomes or modified exosomes.

**Table 1:** Types of EVs and NVEPs.

Name	Category	Size (nm)	Most Referred markers	Site of biogenesis	REF
EVs					
**Exosomes**	Small EV	30–180	CD63, CD81, TSG101, CD9, ALIX, HSP70, HSP90, Alix, Rab5a/b, Syntenin-1	Multivesicular body	[[50], [51], [52]]
**Small Ectosome**	Small EV	30–150	ARF6, LAMP-1, VAMP3, Flotillin-1, β1 integrins, Selectins-1, CD40, MMP	Plasma Membrane	[52,53]
**Microvesicles**	Small/Large EV	150–800	Phosphatidylserine, Annexins A1 and A2, Sphingolipids, and Phosphatidic Acid	Plasma Membrane	[54]
**Apoptotic Bodies**	Small/Large EV	100–5000	Annexin V, Thrombospondin, C3b complement protein	Apoptosis	[52,55]
**Exophers**	Large EV	3500–4000	Ubiquitinated proteins, LC3, p62, TOM20, proteasome components, and LAMP1, Huntingtin, Tau Protein	unknown	[56]
**Oncosomes**	Large EV	1000–10000	MET, EGFR, mutant KRAS, integrins, Vimentin, AR, MYC, Caveolin-1, CD44, MMP2, and MMP9.	Plasma Membrane	[57,58]
**Migrasome**	Large EV	500–3000	TSPAN4, Integrin alpha 5, Integrin bet 1, Mitofilin	Unknown	[59,60]

NVEPs					
**Exomeres**	NVEPs	28–50	Aldolase A, Enolase 1, HSP90, and Glypican-1, FASN, ACLY	Unknown	[61]
**Supermeres**	NVEPs	22–32	CD63, CD9, CD81, HSP70, HSP90, sphingomyelin, cholesterol, enolase, aldolase, galectin-3, IL-10, and integrin β1	Unknown	[62]
**Lipoprotein**	NVEPs	5–1200	ApoB, ApoA-I, ApoC-II, ApoE, cholesterol, triglycerides, phospholipids, LCAT and LPL, LDL receptor	Exocytosis/plasma membrane assembly	[63,64]
**Viral particles**	NVEPs	30–300	Env, Gag (HIV-1), Spike protein S1 (SARS-CoV-2), Hexon (Adenovirus), VP1 (Polyomavirus)	Plasma membrane, exocytosis, cell lysis	[65]
**Vault**	NVEPs	70	MVP, vPARP, TEP1	Unknown	[66]
**Supramolecular Attack particles**	NVEPs	120	Thrombospondin 1, Perforin 1, Granzyme B	Secretory granules	[67]

FASN, fatty acid synthase; HSPA13, heat shock protein family A (Hsp70) member 13; TGFBI, transforming growth factor beta-induced, ACLY, ATP citrate lyase; Apo, apoprotein; ARF6, ADP ribosylation factor 6; NVEPs: Non-Vesicular Extracellular Particles, SARS-CoV-2, severe acute respiratory syndrome-coronavirus 2; VLDL, very low-density lipoprotein, HDL, high-density lipoprotein; IDL, intermediate-density lipoprotein; LAMP-, Lysosome-Associated Membrane Protein 1; VAMP3, Vesicle-Associated Membrane Protein 3; MMP, Matrix Metalloproteinase; TSPAN4, Tetraspanin-4; MVP, Major Vault Protein; vPARP, Vault Poly(ADP-Ribose) Polymerase; TEP1, Telomerase-Associated Protein 1; TOM20, Translocase of the Outer Mitochondrial Membrane 20; TSG101, Tumor Susceptibility Gene 101.

Small EVs, such as exosomes, range from 30 to 180 nm in size and are primarily derived from two intracellular sources: MVBs and the plasma membrane. In contrast, large EVs, comprising MVs and ABs, are distinguished by their larger size. MVs, ranging from 150 to 800 nm, form via outward budding and shedding of the plasma membrane, a process known as ectocytosis [41,42]. Conversely, apoptotic bodies, typically sized from 100 to 5000 nm, arise during apoptosis, characterized by the fragmentation of apoptotic cells. However, some MVs, as well as apoptotic bodies, may fall within the size range of small EVs [44]. These ABs encapsulate cellular debris and organelles, facilitating the clearance of apoptotic material by neighboring cells and phagocytes [[45], [46], [47]]. In addition to these well-characterized EV types, emerging entities such as oncosomes (cancer cell-derived EVs) [48] and supermeres (non-vesicular extracellular particles) have recently been discovered, expanding the scope of the EV landscape [49]. While not traditionally classified within the ISEV's primary EV categories, their biological significance is gaining attention.

### 2.3 Uptake

**Fig. 2:**
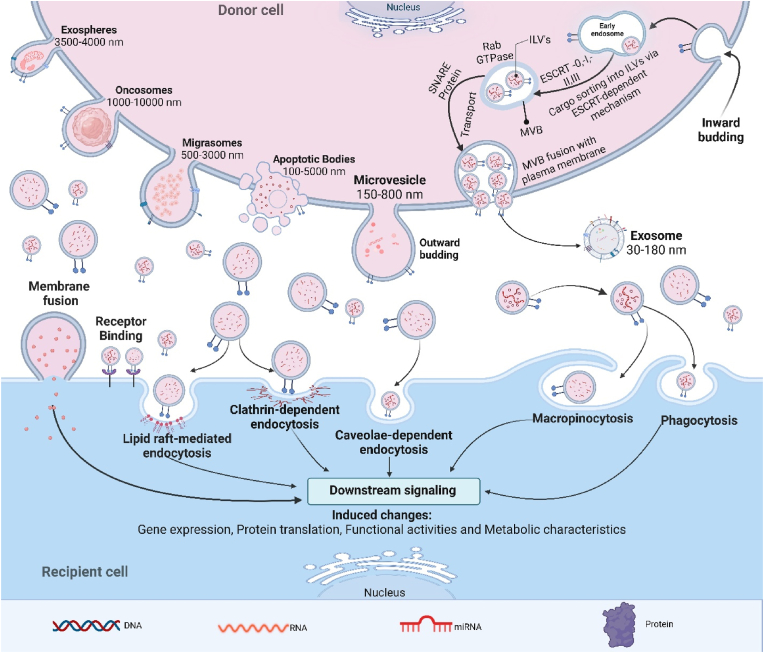
**EVs characteristics, biogenesis, and uptake.** In donor cells, EVs are formed through a process where endosomes bud inward to create multivesicular bodies (MVBs). Within MVBs, small intraluminal vesicles (ILVs) are generated through two distinct pathways. The first pathway involves the ESCRT (Endosomal Sorting Complex Required for Transport)-dependent mechanism, where a series of ESCRT complexes (ESCRT-0, -I, -II, -III) and associated proteins (such as TSG101, ALIX, HRS, and VPS4) facilitate membrane budding and ILV formation. Alternatively, the ESCRT-independent pathway occurs in tetraspanin-enriched microdomains (TEMs) and lipid rafts, where tetraspanins (e.g., CD9, CD81, CD63) aid in cargo sorting and incorporation into ILVs. The release of ILVs as EVs into the extracellular space occurs when MVBs fuse with the plasma membrane, a process mediated by small GTPases (RhoA, Ral-1) and soluble N-ethylmaleimide-sensitive factor attachment protein receptors (SNARE) proteins. This fusion event results in the secretion of EVs, which can then participate in various biological functions, including tissue repair and immune modulation. EVs carry a diverse cargo comprising proteins, DNA, mRNA, and miRNA. They execute their functions by entering recipient cells through various mechanisms, including endocytosis, micropinocytosis, phagocytosis, or direct fusion with the cell membrane. In contrast, microvesicles directly originate from the plasma membrane, while apoptotic bodies are formed through membrane blebs of cells undergoing apoptosis.

EV uptake by recipient cells involves specific interactions between EV surface ligands and cell membrane receptors, facilitating targeted delivery and subsequent cellular responses [68]. The binding of EVs to target cells is influenced by their membrane composition, which includes proteins, lipids, and glycans. Entry mechanisms for EVs include direct membrane fusion, similar to viral entry, where the EV membrane fuses with the recipient cell membrane [69]; endocytosis, which can be clathrin-dependent or clathrin-independent, such as caveolin-mediated uptake and lipid raft-mediated internalization; macropinocytosis, which preferentially internalizes smaller EVs; and phagocytosis, a method by which larger EVs or those flagged for engulfment are internalized (Fig. 2). However, EVs with CD47 can evade this process by signaling ‘do not eat me’ to phagocytic cells like monocytes and macrophages [70]. Post-internalization, EVs release their cargo—proteins, nucleic acids, and lipids—into the recipient cell cytoplasm, influencing gene expression, signaling pathways, and cellular functions [71,72]. However, another potential fate of internalized EVs is lysosomal degradation. Following endocytosis, EVs can be trafficked to endosomes, which may mature and fuse with lysosomes, leading to the degradation of their cargo. This pathway is a regulatory mechanism that limits excessive signaling while controlling which cargo escapes lysosomal breakdown to influence cellular processes [73]. Studies have indicated that EVs can either be recycled or directed to lysosomes, depending on factors like EV composition and recipient cell type [74]. Alternatively, EVs can modulate recipient cells through ligand-receptor interactions on the cell surface, which can induce signaling cascades without internalization. For instance, they can activate T-cell receptors [69]. Their size, surface characteristics, and specific molecular interactions with receptors on the recipient cells influence EVs' uptake and functional impact [23].

## 3 Therapeutic application of EVs in IBD

### 3.1 Modulating immune response

MSC-derived EVs offer promising therapeutic potential for IBD through immune modulation [75]. These EVs reprogram macrophages from a pro-inflammatory to an anti-inflammatory phenotype, thereby aiding in inflammation resolution and tissue repair [76]. For example, EVs from hypoxia-primed adipose-derived stem cells (ASCs) derived from C57BL/6J, 10-day-old male mice deliver miR-216a-5p, effectively promoting anti-inflammatory macrophage polarization via the HMGB1/TLR4/NF-κB pathway in dextran sulfate sodium (DSS)–induced mouse colitis [77]. The HMGB1/TLR4/NF-κB pathway regulates inflammation, with HMGB1 (high-mobility group box 1) acting as a damage-associated molecular pattern (DAMP) that triggers TLR4, leading to NF-κB activation and the release of pro-inflammatory cytokines such as TNF-α, IL-1β, and IL-6 [78,79]. EVs derived from hypoxia-primed ASCs deliver miR-216a-5p, effectively suppressing this signaling cascade. This suppression reduces NF-κB activation and promotes a phenotypic shift in macrophages from pro-inflammatory (M1) to anti-inflammatory (M2) states, which are associated with tissue repair and inflammation resolution [80]. The ability of these EVs to modulate immune responses suggests that hypoxia-primed ASCs can be strategically utilized to manage inflammation in IBD, offering a potential therapeutic approach that leverages the delivery of miR-216a-5p to dampen excessive inflammation and promote healing [77]. Liao et al. demonstrated that Treg-derived EVs (Treg-EVs) alleviate DSS-induced IBD by transferring miR-195a-3p, which regulates Caspase 12, a pro-apoptotic protein implicated in IBD [81]. The reduction in Caspase 12 protein levels in colon tissues of IBD mice was reversed when miR-195a-3p expression in Treg-EVs was downregulated. Their results suggest that miR-195a-3p′s therapeutic potential in IBD is mediated through its inhibition of Caspase 12, highlighting its promise as a targeted treatment.

**Fig. 3:**
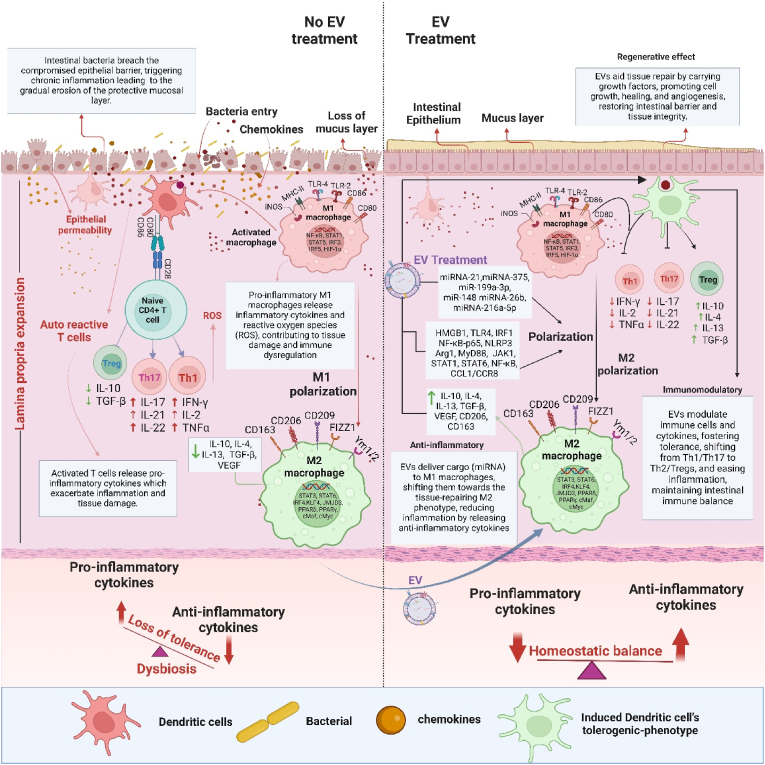
EVs exert a therapeutic effect by restoring intestinal integrity. A compromised epithelial barrier initially permits bacterial infiltration, likewise, bacteria EVs, triggering inflammation and mucosal erosion. Following EV treatment, macrophages display reduced pro-inflammatory cytokine release, increased anti-inflammatory cytokine production, and a shift toward M2 polarization. Likewise, dendritic cells transition to a tolerogenic phenotype in response to EV exposure, promoting regulatory T cell generation and suppressing pro-inflammatory cytokines, thereby effectively modulating the immune response.

In addition to their effects on macrophages, MSC-derived EVs also transfer miRNAs that suppress T-cell proliferation and cytokine production, thereby mitigating the exaggerated immune response seen in IBD [82]. These vesicles modulate T-cell differentiation towards Tregs, essential for promoting immune tolerance and reducing inflammation [83]. (Fig. 3). In experimental colitis models using C57BL/6 mice, EVs derived from olfactory ecto-mesenchymal stem cells (eMSC-EVs) have shown significant immunosuppressive effects, mainly by inhibiting the differentiation of T helper 1 (Th1) and T helper 17 (Th17) cells while promoting Treg cell induction [84]. Th1 cells, which produce pro-inflammatory cytokines like IFN-γ and TNF-α, are critical drivers of inflammation through the activation of macrophages and enhanced production of reactive oxygen species (ROS). Th17 cells, characterized by their secretion of interleukin-17 (IL-17), recruit neutrophils and produce additional pro-inflammatory cytokines, such as IL-6 and IL-1β, which exacerbate tissue damage and intestinal inflammation [85]. Specific cytokines and transcription factors regulate both Th1 and Th17 differentiation pathways: Th1 cells are driven by IL-12 and T-bet through the STAT4 pathway, whereas Th17 cells are promoted by TGF-β, IL-6, and IL-23, engaging the RORγt and STAT3 pathways [86]. The eMSC-EVs potentially modulate these pathways by delivering bioactive molecules, including miRNAs and proteins, that suppress the expression of key transcription factors like T-bet and RORγt, thereby reducing the levels of IFN-γ and IL-17. This suppression curtails the recruitment and activation of additional pro-inflammatory cells, alleviating inflammation [72]. Simultaneously, eMSC-EVs promote Treg cell induction, enhancing the production of anti-inflammatory cytokines such as IL-10 and TGF-β, further supporting immune homeostasis and tissue repair [84]. Moreover, EVs isolated from breast milk have demonstrated similar immunomodulatory effects, promoting Treg cell proliferation dose-dependently, likely by enhancing TGF-β signaling and FoxP3 expression, crucial for Treg differentiation [87]. Additionally, human umbilical cord-derived MSC (hUC-MSC)-EVs alleviate DSS-induced colitis in mice by downregulating inflammation-associated genes such as TNF-α, IL-1β, IL-6, iNOS, and IL-7 while upregulating the anti-inflammatory IL-10 gene in colon tissues [88]. These findings highlight the therapeutic potential of EVs in modulating immune responses, offering a targeted approach to managing IBD by inhibiting pro-inflammatory pathways and fostering immune tolerance. In this regard, EVs have shown considerable potential for modulating the immune response in IBD, as summarized in Table 2, Table 3.

**Table 2:** Immunomodulatory effects of Extracellular Vesicles on Ulcerative Colitis.

EV Sources	Isolation Method	Experimental Model	EV's Conc.	Grouping	Administration Route	Functional cargo	Downstream signaling	Downstream Genes	Upstream Genes	Effects	Ref
BM-MSCs	Ultra-C	***In Vitro*** (LPS-treated Mouse Peritoneal macrophages)	100 μg/mL EVs for 1 day	NA		/	JAK1/STAT1/STAT6	VEGF-A, IFN-γ, IL-12, TNFα, CCL-24, and CCL-17 downregulation	IL-10 and TGF-β levels upregulated	There was a reduction in weight loss, DAI, and colon mucosa damage and an increase in colon length. EVs promoted M2-like macrophage polarization, characterized by the increased M2 markers CD163 and CD200R.	[89]
		Mice DSS-induced Colitis	50 μg/mL EVs for 7 days	(n = 10 per group)	IP						
Bovine colostrum	Ultra-C	***In Vitro*** (NCM460 and RAW264.7 cells LPS-treated macrophages)	0.1 mg ml^−1^ EVs for 1 day	NA		miRNA-21, miRNA-375 and miRNA-26b	/	TNFα, IL-6, ROS and, iNOS downregulation	IL-10 was upregulated	Col-EVs alleviated colitis symptoms, including weight loss, gastrointestinal bleeding, and chronic diarrhea, by modulating intestinal inflammatory immune responses.	[90]
		Mice DSS-induced Colitis	50 mg kg−1 B W Col-Evs for 7 days	(n = 5 per group)	OG						
CbEVs	Ultra-C	***In Vitro*** (RAW264.7 cell LPS-treated macrophages)	1 μg/mL CbEVs for^1/6^ a day.	NA		miR-199a-3p	MAPK and NF-*κ*B	Tlr4, Nf-kb, TNf-*α*, F4/80, Cd11c, Mcp1, and Ccl-5 downregulated	TRP, ILA, and IA upregulated	CbEVs attenuated intestinal inflammation by Modulating intestinal miRNAs.	[91]
		Sprague–Dawley Rats DSS-induced Colitis	DSS_CbEVs, 50 μg day^−1^	(n = 8 per group)	OG					Restored microbial balance by decreasing the abundance of pathogenic species such as *E. coli* and *S. flexneri*.	
AD-MSCs	Ultra-C	Sprague–Dawley Rats DSS-induced Colitis	100 μg/mL EVs for 7 days	(n = 7 per group)	IP	/	/	IFN-γ, TNF-α, IL-12, and IL-17 downregulated	TGF-β, IL-4, Treg cell, and IL-10 upregulated	Diminished colon shortening, body-weight loss, bleeding, and colon injury.	[92]
*Lactobacills plantarum* Q7	Ultra-C	Sprague–Dawley Rats DSS-induced Colitis	(0.5 mg/kg BW, daily)	NA	OG	/	TLR4-MyD88-NF-kB	IL-6, IL-1b, IL-2, and TNF-a downregulated	/	Induced colon shortening, bleeding, and body weight loss were observed. Improved the dysregulation of gut microbiota and promoted the diversity of gut microbiota.	[93]
			(1 mg/kg BW daily)								
MCEVs	DC	Sprague–Dawley Rats DSS-induced Colitis	100 μL for 5 days	(n = 5 per group)	OG	Thioredoxin and Peroxidases	NF-*κ*B	IL-1β, IL-6, and TNF-α, LDH and MDA downregulated	GSH, GSH-PX, SOD, CAT, IL-10 upregulated	MCEVs protected the colonic mucosa by regulating the oxidation and inflammation indexes, and the symptoms of colonic ulceration were alleviated.	[94]
CBEVs	Ultra-C	Sprague–Dawley Rats DSS-induced Colitis	15μg/mL^−1^ of EVs	(n = 6 per group)	IG	/	STAT6/Arg1	TNF-α and NOS_2_ downregulated	TGF-βand IL-10 upregulated	Improved the remission of murine colitis and polarized the transformation of macrophages to the M2 type. Restored gut dysbiosis and improved the impact of EVs on the reprogramming of the M2 macrophages	[95]
mEVs	Ultra-C	Sprague–Dawley Rats DSS-induced Colitis	0.6, 1.8, and 3.0 mg/kg/day).	(n = 5 per each group).	OG	miR-148	TLR4-NF-κB and NLRP3	iNOS, COX2, IL-1β, TNF-α, IL-6, IL-2, and IL-22 downregulated	IL-10 upregulated	Attenuation of intestinal inflammation.	[96]
										Restoration of Treg/Th17 cell balance. Modulation of gut microbiota composition	
FhEVs	Ultra-C	Sprague–Dawley Rats DSS-induced Colitis	10 μg of FhEVs on days 0, 15, and 30	(n = 3 Per each group).	SI	/	MAPK and NF-kB	TNFα, COX-2, IL-6, and IL17A downregulated	/	Reduce pro-inflammatory cytokines. Exert effects independent of T-cells, suggesting involvement of other immune cell types.	[97]
BM-MSCs	Ultra-C	Male Sprague–Dawley rats TNBS)-induced colitis model.	(50 μg 100 μg and 200 μg EV)	(n = 10 per each group).	IV	/	NF-κBp65	TNFα, iNOS, COX-2, IL-1β, MPO and MDA downregulated	IL-10, SOD, and GSH upregulated	Reducing the cleavage of caspase-3, caspase-8, and caspase-9 can modulate inflammation, suppress oxidative stress, and alleviate apoptosis.	[98]
LGG-EVs	Ultra-C	Sprague–Dawley Rats DSS-induced Colitis	1.2 mg/kg LGG-EVs BW for 14 days	(n = 10 per each group).	OG	/	TLR4-NF-κB-NLRP3	TNF-α, IL-1β, IL-6, and IL-2 downregulated	/	Attenuation of intestinal inflammation.	[99]
										Reduce pro-inflammatory cytokines. Improved the dysregulation of gut microbiota.	
TD-EVs	Ultra-C	Sprague–Dawley Rats DSS-induced Colitis	3mg/dose for 7 days	(n = 5 per each group).	OG	/	NF-κB-p65	TNF-α, IL-6, and IL-1β downregulated	HO-1 upregulated	Ameliorate and improve the resolution of colitis by regulating the pro-inflammatory expression.	[100]
B-mEVs Hu-mEVs	Ultra-C	***In Vitro*** Macrophages cell RAW264.7	0.6, 3.0 mg/kg per day for 7 days	/		/	/	TNFα, CD40 and IL-6 downregulated	ZO-1and Occludin Upregulated	Decreased disruption of the colonic epithelium, colonic fibrosis, and inflammatory cell infiltration.	[101]
		Mouse dendritic cell line								Increased expression of tight-junction proteins and mucins.	
		Epithelial Caco-2 cells								Decreased weight loss, decreased activity scores, decreased colon shortening.	
		Mice DSS-induced Colitis		(n = 3 per each group)	OG						
PD-L1-EVs	Ultra-C	Sprague–Dawley Rats DSS-induced Colitis	0.4 mg/kg/Day	(n = 10 per each group).	IP	/	PI3K/Akt/mTOR	IFN-γ, IL-1β, IL-8, IL-6, IL-2, BAX, NF-κB, TNF-α, MPO, and MDA downregulated	IL-4, BCL-2, SOD, PTEN and GSH upregulated	Reduced inflammation, cell death, and oxidative stress in the colon.	[102]
										Modulated the equilibrium of Th17/Treg cells.	
MenSCs-sEV	Ultra-C	Sprague–Dawley Rats DSS-induced Colitis	200 μg/mL EVs for 7 days	(n = 6 per each group).	IP	/	IRF1	TNF-α, IFN-γ, IL-1β, and IL-6 downregulated	IL-10, ZO-1, ZO-2 and Occludin Upregulated	Decrease inflammation in the colon. Promote polarization of M2 macrophages in the colon.	[103]
AD-MSCs	Ultra-C	Sprague–Dawley Rats DSS-induced Colitis	200 μg/mL EVs for 3 days	(n = 5 per each group).	IP	miR-216a-5p	HMGB1/TLR4/NF-κB	iNOS, TNF-α, IL-1β, and IL-6 downregulated	Arg1 and CD206 upregulated	Promote macrophage M2 polarization.	[77]
M2B-EVs	Ultra-C	Sprague–Dawley Rats DSS-induced Colitis	50 mg for 8 days	(n = 5 per each group).	IP	/	CCL1/CCR8	IL-1β, IL-6, and IL-17A downregulated	IL-4 Upregulated	Reduce colon length and damage	[104]
										Modulated the equilibrium of Th17/Treg cells.	

IP: Intraperitoneal injection, IG: Intragastrically, OG; Oral gavage, SI: Subcutaneous Injection, IV: Intravenous injection, BM-MSC: Bone-Marrow Mesenchymal Stromal cells, AD-MSC: Adipose Tissue Mesenchymal Stromal Cells, CbEvs: Clostridium butyricum, mEVs: Milk-derived, MenSCs-sEV: Human Menstrual blood-derived Mesenchymal Stromal cells small EVs, Hu-mEVs: Human Milk extracellular Vesicle, B.MEVs: Bovine Milk Extracellular Vesicle, M2B-EV: Macrophages Derived EVs/FhEVs: Fasciola hepatica, TDEVs: Tumeric Derived EVs, MCEVs: Momordica charantia-derived extracellular vesicles, LGGEVS: Lactobacillus rhamnosus GG, PD-L1-EVs: Programmed death-ligand 1, Ultra-C: Ultracentrifugation, DC: Differential Centrifugation, LPS: Lipopolysaccharide, DSS: Dextran Sulfate Sodium.

**Table 3:** Immunomodulatory effects of Extracellular Vesicles (EV) on Crohn's Disease.

EV Sources	Isolation Method	Experimental Model	EV's Conc.	Grouping	Administration Route	Functional cargo	Downstream signaling	Downstream Genes	Upstream Genes	Effects	Ref
AD-MSCs	Ultra-C	***In Vitro*** (THP-1 cell PMA-treated macrophages)	50 μg	(n = 3)	NA	/	JAK-STAT, MEK/ERK	TNF-α, IL-6, and IL-12, downregulated	/	A Pro-inflammatory state was observed. Healthy MSCs reverse inflammation by inducing the M2 monocyte subset.	[105]
mEVs	Ultra-C	***In Vitro*** (IPEC-J2 cells LPS-treated macrophages)	0.6 μg mEVs for 2 days	NA	NA	/	TLR1	NOS2, MMP9, TLR5, TGFB1, IFNB, IL-18 and IL-12A, downregulation	DEFB, TLR1, 2 upregulated	Modulate the expression of TLRs and cytokines in swine intestinal cells. Attenuation of intestinal inflammation.	[106]

mEVs: Milk-derived, AD-MSC: Adipose Tissue Mesenchymal Stroma cells.

## 4 Tissue repair and regeneration

EVs are emerging as transformative tools in tissue repair, delivering therapeutic payloads to damaged tissues [107]. Recent research highlights their substantial potential in preclinical and clinical settings (Table 4). EVs derived from MSCs exhibit a unique protein and transcript profile that supports vasculogenesis and angiogenesis, primarily through modulation of the NF-κB signaling pathway [108]. Key miRNAs in MSC-derived EVs, such as miR-21, miR-23a, miR-125b, and miR-145, play a crucial role in reducing scar formation by inhibiting TGF-β2/SMAD2 signaling, which has implications for IBD management [109]. Besides this, EVs from bone marrow-derived MSCs (BMSCs) significantly reduced colonic inflammation in tri-nitrobenzene sulfonic acid (TNBS) IBD mice models [92]. This effect was achieved by downregulating the expression of pro-inflammatory cytokines, including IFNγ, TNF-α, IL-12, IL-1β, CCL-17, and CCL-24, while upregulating the anti-inflammatory cytokines, such as IL-10 and TGF-β levels [92]. Additionally, *in vitro* studies with lipopolysaccharide (LPS)-treated macrophages show that EVs can attenuate colitis by promoting M2-like macrophage polarization via the JAK1/STAT1/STAT6 signaling pathway [89].

**Table 4:** Tissue Repair and Regeneration effects of Extracellular Vesicles (EVs) application on Inflammatory Bowel Disease.

EV Sources	Isolation Method	Experimental Model	EV's Concentration	Grouping	Administration Route	Functional cargo	Downstream signaling	Downstream Genes	Upstream Genes	Effects	Ref
Huc-MSC-Evs-derived TSG6	UC	Mice DSS-induced Colitis	200 μg/mL after a day	(n = 5 per group)	IP	/	/	Th17 downregulated	Th2 Upregulated	Restore intestinal immunological homeostasis and the mucosal barrier to prevent IBD.	[114]
MSC-Evs	UC	Mice DSS-induced Colitis	200 μg/mL	(n = 20 per group)	IV	metallothionein-2	NF-κB	IL-1β, IL-6, and MPO, TNF-α downregulated	IL-10 upregulated	Diminish colonic inflammation in mice by a mechanism that depends on macrophages.	[115]
BM-MSCs	UC	Mice DSS-induced Colitis	0.4 mL Evs	(n = 5 per group)	IV	/	/	D-LAC, DAO, MDA downregulated	SOD upregulated	Mucosal permeability boost Repairing the damaged digestive tract Reduction of oxidative stress in patients with colitis	[116]
BM-MSCs	UC	Mice DSS-induced Colitis	200 μg/mL	(n = 5 per group)	IP	/	/	TNF-α downregulated	IL-10, Treg and TGF-β upregulated	Reduction of inflammatory cytokines Defense against harm to the crypt structure	[117]
AD-MSCs	UC	Mice TNBS-induced colitis model.	(0.5 mL)	(n = 20 per group)	IV	miR-1236	/	RORy, MDA, MPO, and NO downregulated	/	Reduction in the inflammation indicators Restored mucosal damage	[118]
hUC-MSCs	UC	Mice DSS-induced Colitis	NA	NS	IV	/	NOD2–RIP2	IL-6, IFN-γ, PGE_2_ and TNF-α downregulated	IL-10, Treg and TGF-β upregulated	Reduction of submucosal edema and mucosal damage Reduction of colon inflammation Increase in colon tissue Treg infiltration	[119]
TN-MSCs	UC	Mice DSS-induced Colitis	(1 × 10^6^ cells/mouse/day) on days 6 and 16	(n = 9 per group)	OG	/	/	IL-1β, IL-6, and IL-17, TNF-α downregulated	/	Colon length normalization Decreases DAI scoring	[120]
BM-MSCs	UC	Mice DSS-induced Colitis	50 μg/mL EVs for 7 days	(n = 10 per group)	IP	/	JAK1/STAT1/STAT6	VEGF-A, IFN-γ, IL-12, TNFα, CCL-24, and CCL-17 downregulation	IL-10 and TGF-β levels upregulated	Reduction in weight loss, DAI, and colon mucosa damage. Increase in colon length.	[89]
AD-MSCs	UC	Mice DSS-induced Colitis	100 μg/mL EVs for 7 days	(n = 7 per group)	IP	/	/	IFN-γ, TNF-α, IL-12, and IL-17 downregulated	TGF-β, IL-4, Treg cell, and IL-10 upregulated	Diminished colon shortening, bleeding, and colon injury.	[90]
*Lactobacills plantarum* Q7	UC	Mice DSS-induced Colitis	(0.5 mg/kg BW, daily) (1 mg/kg BW daily)	NA	OG	/	TLR4-MyD88-NF-kB	IL-6, IL-1b, IL-2, and TNF-a downregulated	/	Induced colon shortening and bleeding were observed.	[93]
B-mEVs Hu-mEVs	UC	Mice DSS-induced Colitis	0.6, 3.0 mg/kg per day for 7 days	(n = 3 per each group)	OG	/	/	TNFα, CD40 and IL-6 downregulated	ZO-1and Occludin Upregulated	Decreased disruption of the colonic epithelium, colonic fibrosis, and inflammatory cell infiltration. Increased expression of tight-junction proteins and mucins. Decreased weight loss, decreased activity scores, decreased colon shortening	[102]
MenSCs-sEV	UC	Mice DSS-induced Colitis	200 μg/mL EVs for 7 days	(n = 6 per each group).	IP	/	IRF1	TNF-α, IFN-γ, IL-1β, and IL-6 downregulated	IL-10, ZO-1, ZO-2 and Occludin Upregulated	Decrease inflammation in the colon. Promote polarization of M2 macrophages in the colon.	[103]
M2B-EVs	UC	Mice DSS-induced Colitis	50 mg for 8 days	(n = 5 per each group).	IP	/	CCL1/CCR8	IL-1β, IL-6, and IL-17A downregulated	IL-4 Upregulated	Reduce colon length and damage	[104]

IP: Intraperitoneal injection, OG; Oral gavage, IV: Intravenous injection, UC: Ultracentrifugation, hUCMSC: human umbilical cord MSC-EVs, BM-MSC: Bone-Marrow Mesenchymal Stem Cells, AD-MSC: Adipose Tissue Mesenchymal Stem Cells, NA: Not Available, IP: Intraperitoneal injection, OG; Oral gavage, IV: Intravenous injection, DSS: Dextran Sulfate Sodium, TNBS: 2,4,6-trinitrobenzene sulfonic acid.

Song et al. demonstrated that EVs from hUC-MSCs induce M2 macrophage polarization in the IBD mouse model induced by TNBS and DSS. This shift towards M2 macrophages was associated with reduced colonic inflammation and enhanced mucosal healing, highlighting the ability of hUC-MSC-derived EVs to modulate immune responses and promote tissue regeneration [110]. Complementing these findings, Li et al. investigated EVs from human ASCs in a critical-sized calvarial defect model using a PLGA/pDA scaffold. Their study found that hASC-derived EVs significantly enhanced angiogenesis and bone regeneration by delivering bioactive molecules that stimulate endothelial cell proliferation and osteoblast activity, demonstrating their potential for clinical applications in bone repair [111]. Additionally, Yu et al. explored the effects of EVs from EphB2-overexpressing bone marrow MSCs (EphB2-EVs) in DSS-induced colitis, revealing that EphB2-EVs reduce intestinal mucosal inflammation and accelerate colon tissue repair. This effect was linked to the modulation of immune cell populations, explicitly rebalancing Th17 and Treg cells and inhibiting STAT3 activation. EphB2-EVs also restored the intestinal mucosal barrier and reduced oxidative stress, indicating their promise as a novel approach for treating chronic intestinal inflammation [112].

Yang et al. examined rat models of intestinal fibrosis induced by TNBS. They discovered that EVs carrying miR-200b could ameliorate colonic fibrosis by inhibiting the epithelial-to-mesenchymal transition (EMT), as well as restoring the morphology of intestinal epithelial cells (IECs) by downregulating vimentin and smooth muscle actin and upregulating E-cadherin expression. Ultimately, this modulated colon EMT by targeting the fibrosis-related proteins ZEB1 and ZEB2 [113]. The modulation was accomplished by downregulating protein expression, specifically those that function as transcriptional repressors of E-cadherin. Moreover, their research emphasized the capacity of EV-encapsulated RNA and proteins to operate as endogenous molecules once delivered to recipient cells.

## 5 Drug delivery system in IBD

**Fig. 4:**
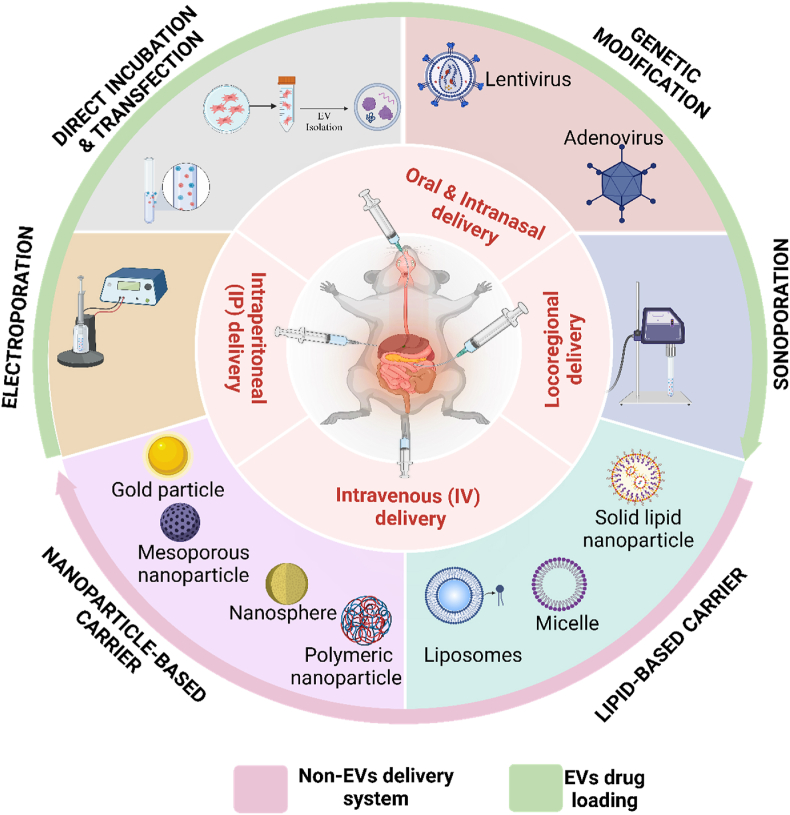
This diagram illustrates various methods for loading EVs with therapeutic cargo and nanoparticle drug delivery. Direct Incubation involves mixing EVs with the cargo, allowing passive diffusion. Genetic Modification uses viral vectors like lentivirus and adenovirus to introduce genetic material into EVs. Sonoporation employs ultrasound waves to create temporary pores in the EV membrane for cargo entry. Electroporation uses an electrical field to permeabilize the EV membrane, facilitating cargo loading. Lipid-based Carriers, such as liposomes and solid lipid nanoparticles, encapsulate the cargo and merge with EVs to enhance loading. Nanoparticle-based Carriers involve gold particles or polymeric nanoparticles to deliver cargo into EVs. The central illustration depicts various delivery methods: Oral & Intranasal, Intraperitoneal (IP), Intravenous (IV), and Locoregional delivery.

Nanoparticles and EVs represent innovative platforms for drug delivery, offering distinct advantages in targeted and controlled drug administration, particularly in the challenging landscape of intestinal delivery. As drug delivery systems, nanoparticles leverage various encapsulation techniques, including lipid-based carriers and sequential nano-precipitation (Fig. 4) [121]. These approaches are integral in achieving high drug-loading capacities while preserving the pharmacological activity of the cargo. The lipid-based carriers, in particular, provide a biocompatible interface, closely mimicking natural cell membranes, facilitating more accessible fusion and penetration into target cells. This characteristic is precious in crossing the intestinal epithelial barrier, where tight junctions often limit the absorption of macromolecular drugs. Using sequential nanoprecipitation, nanoparticles can be synthesized with controlled size distribution, optimized drug release profiles, and enhanced encapsulation efficiency. This precision in nanoparticle synthesis directly correlates with improved drug pharmacokinetics, where controlled release mitigates burst effects and enhances therapeutic concentrations at the target site [122].

On the other hand, EVs present a natural and biological advantage due to their tissue-specific receptors and innate ability to fuse with target cells and deliver cargo. Unlike nanoparticles, which lack tissue-specific homing strategies and surface receptors, EVs naturally express tissue-specific receptors, enabling them to target and fuse with recipient cells precisely [123]. This fusion allows EVs to deliver their cargo directly into the cytoplasm, facilitating the transfer of biomolecules such as proteins, RNAs, and microRNAs. Additionally, EVs can engage with signaling pathways by binding to receptors on the surface of recipient cells, activating downstream cellular responses. In contrast, although effective for packaging and delivering therapeutic agents, nanoparticles do not possess these receptor-mediated capabilities and cannot activate signaling pathways. Moreover, as foreign entities, nanoparticles are more likely to trigger immune responses, limiting their efficacy [124]. EVs' biocompatibility and natural targeting abilities make them superior for therapeutic applications where specific delivery and minimal off-target effects are essential [125].

EV Drug-loading strategies include sonoporation, electroporation, genetic modification, and direct incubation [126] (Fig. 4) enable the stable encapsulation of drugs without compromising the EV membrane structural integrity, which is critical in maintaining their bioactivity. Sonoporation and electroporation allow for the transient disruption of EV membranes, creating pores that facilitate drug entry without inducing structural damage. This ensures the encapsulated drugs remain intact during the delivery process, a critical consideration for therapies involving fragile molecules like mRNA or siRNA [127].

A notable application of genetic modification and direct incubation is seen in studies where EVs engineered to carry methotrexate and triptolide significantly reduced drug toxicity while enhancing therapeutic effects in IBD models [125]. The reduction in toxicity is attributed to the EVs' targeted delivery capabilities, which enable precise localization of the drug at the site of inflammation, thereby minimizing off-target effects commonly observed with systemic drug administration [128]. This represents a shift towards safer therapeutic options, particularly in treating diseases that require long-term drug administration, such as IBD. The ability to avoid systemic exposure, particularly with potent compounds like triptolide, is crucial in preventing adverse effects, such as immunosuppression or organ toxicity, which often limit the use of such agents in clinical settings [125].

The therapeutic potential of EVs is further highlighted in their capacity to be genetically engineered to deliver specific molecular modulators. For instance, BMSCs transfected with lentiviruses to overexpress miR-146a were found to significantly suppress inflammation by downregulating TNF receptor-associated factor 6 (TRAF6) and IL-1 receptor-associated kinase 1 (IRAK1) in colitis models [129]. miR-146a is a crucial regulator of the NF-κB pathway, a central player in inflammatory responses, which is often dysregulated in chronic inflammatory diseases like IBD. By inhibiting this pathway, miR-146a-loaded EVs offer a precise method of dampening inflammation at the molecular level [130]This regulatory effect on NF-κB highlights the broader potential of miRNA-based therapies in providing targeted treatments for inflammatory diseases. They offer a more refined approach than traditional anti-inflammatory drugs, which often have broad systemic effects.

Similarly, the overexpression of miR-200b through genetic modification-induced passive loading in BMSC-derived EVs has been shown to prevent EMT in TGF-β1-treated IEC-6 cells, a critical process in the development of fibrosis in chronic intestinal diseases [131]. miR-200b targets ZEB1 and ZEB2, transcription factors known to induce EMT, which plays a pivotal role in tissue remodeling and fibrosis [132]. The ability of miR-200b-loaded EVs to reverse EMT improves the morphological characteristics of affected tissues and restores normal function, as demonstrated by improved histological outcomes in TNBS-induced colon fibrosis models. The suppression of EMT presents a vital therapeutic strategy in preventing long-term complications of IBD, such as stricture formation, which arises due to excessive fibrotic tissue deposition.

Further genetic modifications to EVs, such as M2 macrophage-derived EVs (M2-EVs) carrying LncRNA MEG3, have been shown to modulate inflammatory pathways via the miR-20b-5p/CREB1 axis [133]. CREB1 is a transcription factor involved in cellular responses to stress and inflammation, and its modulation through MEG3-overexpressing EVs represents a novel approach to restoring homeostasis in UC models [134]. The clinical implications are profound, as such therapies could offer an alternative to immunosuppressive treatments, often associated with significant adverse effects. By providing a localized, cell-type-specific modulation of immune responses, MEG3-loaded M2-EVs could offer a targeted therapeutic option that reduces the need for broad-spectrum immunosuppression in patients with UC [135].

In another study, Gal-IL10-EVs demonstrated the ability to control ROS production and inhibit pro-inflammatory cytokine expression while simultaneously improving colonic barrier function [136]. IL-10 is a crucial anti-inflammatory cytokine, and its incorporation into EVs through genetic modification-induced loading provides a stable, localized delivery system that enhances its therapeutic efficacy. The ability of these EVs to traverse the intestinal barrier and deliver bioactive proteins directly to inflamed tissues is of particular significance, as it offers a novel route for protein-based therapies in gastrointestinal diseases. Such an approach could be precious for patients with severe IBD, where traditional systemic treatments fail to control inflammation or restore intestinal barrier integrity adequately. Also, milk-derived EVs loaded with anti-TNFα siRNA through electroporation highlight the potential for oral nucleic acid therapies in treating IBD [137]. TNFα is a well-established target in IBD treatment, and the ability to deliver siRNA in a stable, bioavailable form via milk EVs represents a significant advancement in gene therapy approaches [138]. The natural stability of milk EVs within the gastrointestinal tract allows them to protect siRNA from enzymatic degradation and effectively deliver it to the colon.

## 6 Clinical applications of EVs in IBD

A few clinical studies are currently in progress to assess the safety and efficacy of EV-based therapeutics in IBD (Table 5). Phase 1 clinical research evaluated the safety and viability of MSC-derived EVs (Exo-Flo) treatment in patients with refractory Crohn's disease. In this study, an intravenous infusion of Exo-Flo was administered to assess side effects and possible therapeutic benefits [NCT05836883]. Despite the encouraging results from current clinical studies, the development and use of EV-based therapeutics for immune-mediated disorders still face several obstacles. Addressing safety issues, upscaling large quantities, standardizing isolation and characterization procedures, and optimizing dosage schedules are crucial to advancing the field. Further study is required to clarify the mechanisms through which EVs exert their therapeutic benefits and to determine the best course of action for various disease situations.

**Table 5:** Clinical trials of extracellular vesicles in IBD therapeutic applications.

Title of Study	Diseases Type	EV Sources	Vol.	Study Type	Intervention Model	Number of Subjects	Criteria	Trial Phase	Status	Delivery Route	Country	Objectives	Duration Time	Ref/Clinical trial number
Study of ExoFlo for the Treatment of Perianal Fistulas	Perianal Fistulizing Crohn's Disease	ExoFlo (BM-MSCs)	15 & 30 mL	Interventional	Multicenter, Placebo-controlled, Dose-escalation Design, Randomized controlled trial	12	Men and women between 18 and 75 years of age diagnosed with Crohn's Disease for at least six months were required. They had single and/or multi-tract perianal fistula(s)	IB/IIA	Recruiting	IV	United States of America	Assessing the safety and viability of ExoFlo as a therapeutic option for Perianal Fistulizing Crohn's Disease	12 Months	NCT05836883
Study of ExoFlo for the Treatment of Medically Refractory Crohn's Disease	Crohn's Disease	ExoFlo (BM-MSCs)	15 & 30 mL	Interventional	Single Group Assignment	10	Subjects were required to have had colitis, ileitis, or ileocolitis confirmed previously by radiography, histology, and/or endoscopy at any point in the past. Furthermore, they were mandated to undergo a washout period of at least 8 weeks for any prior monoclonal antibody therapy	I	Recruiting	IV	United States of America	Assessing the feasibility and safety of IV ExoFlo in Medical Refractory Crohn's disease patients	24 Months	NCT05176366
Safety of Injection of Placental MSC-EVs for Treatment of Resistant Perianal Fistula in Crohn's Patients	Perianal Fistulizing Crohn's Disease	Placental MSC-EVs	5 mL	Interventional	Parallel Assignment	40	The study required individuals between 18 and 70 years old to be diagnosed with Crohn's disease. Participation in the study required providing informed consent	I/II	/	IV	Islamic Republic of Iran	Evaluating the safety and efficacy of injected EVs over three months, assessing adverse outcomes, clinical response regarding fistula closure, and changes in inflammatory markers such as CRP, IL-6, TNF-α, and calprotectin.	3 Months	NCT05402748
Study of ExoFlo for the Treatment of Medically Refractory Ulcerative Colitis	Ulcerative Colitis	ExoFlo (BM-MSCs)	15 mL	Interventional	Single Group Assignment	10	Participants were required to have medically refractory ulcerative colitis and were not to have had any previous intestinal surgeries related to ulcerative	I	Recruiting	IV	United States of America	Evaluating the feasibility and safety of intravenous ExoFlo in subjects with medically refractory ulcerative colitis who had failed, were intolerant, or had a contraindication to one or more monoclonal antibodies	24 Months	NCT05176366
Plant EVs±Curcumin to Abrogate Symptoms of IBD	IBD (UC & CD)	Ginger EVs (Curcumin)		Interventional	Parallel Assignment	30	Participants were required to have a confirmed diagnosis of IBD with moderate DA.	IV	Completed		United States of America	Assessing the safety and tolerability of EVs with and without curcumin in patients with IBD and estimating the impact of ginger EVs or curcumin alone or combined with curcumin on symptoms and disease scores in patients with refractory IBD, toxicities associated with ginger EVs. Additionally, the effect of ginger EVs on biomarkers of inflammation was evaluated	1 Month	[139]

## 7 Challenges and future direction

The therapeutic potential of EVs for IBD is intriguing. However, realizing this promise in clinical practice would require overcoming numerous significant difficulties [140]. A major challenge is large-scale EV production, where the ultimate yield, nature, and purity are heavily impacted by the purification and enrichment procedures used [141]. The gold standard for EV research is ultracentrifugation, although it has low scalability and recovery rates [142]. Consequently, additional size-based separation techniques such as flow field-flow fractionation (FFF) and size exclusion chromatography (SEC) are becoming more well-known [143]. From research to therapeutic uses, these methods offer more consistent yields and higher scalability—qualities absolutely essential. While density gradient ultracentrifugation remains the preferred approach for separating EVs from soluble components, the use of affinity-based purification methods has raised concerns regarding the specificity and ubiquity of EV markers [144]. So, while promising, these approaches may increase heterogeneity due to the necessity for globally acknowledged EV markers, affecting the repeatability and standardization of EV-based treatments. Other separation approaches, such as polymer precipitation and ion exchange chromatography, have also been investigated, with each posing distinct scalability, purity, and bioactivity issues [145,146]. There is rising interest in using tactics utilized in other bioparticle production processes, such as chromatography and ultrafiltration, to solve these issues [147]. Previously used to concentrate and purify viral particles, tiny proteins, and antibodies, the same approaches are now being modified for EVs [148]. Implementing these processes in EV manufacturing might dramatically improve scalability and consistency, making large-scale clinical applications more viable.

EV isolation and characterization methods must be standardized absolutely. Their variability much hampers the therapeutic potential of EV populations as various subtypes may show diverse biological roles [122]. Ensuring the consistency of EV preparations used in clinical environments depends on standardizing separation techniques such as differential ultracentrifugation and affinity-based capture and creating thorough content analysis methodologies [149]. By means of machine learning (ML) algorithms, high-dimensional data from proteomics, lipidomics, and transcriptomics could be analyzed to reveal molecular profiles of EVs that match therapeutic outcomes, thus facilitating the identification of ideal EV subtypes for particular clinical uses [150].

Optimizing EV delivery routes to improve treatment effectiveness is another important topic for further study. Whether intravenous, oral, intranasal, or local injection—the delivery path chosen greatly influences EVs' dispersion, stability, and targeting. Every path offers different difficulties; for instance, oral treatment suffers degradation by digestive enzymes and acidic pH, whereas intravenous delivery results in fast clearance by the reticuloendothelial system [151]. Utilizing developments in nanotechnology and bioengineering, EV formulations resistant to enzymatic degradation, extended circulation durations, and increased tissue penetration capacities might be developed [152]. Furthermore, investigating focused delivery techniques, including surface modification of EVs with ligands or antibodies that identify certain receptors on target cells, might increase the specificity and efficacy of EV-based treatments [72]. Fascinatingly, using ML in the optimization route for EV delivery has great chances for enhancing therapeutic results. Large preclinical and clinical research datasets allow ML models to find trends and forecast the most efficient delivery tactics depending on elements such as EV subtype, cargo content, and patient-specific traits. This data-driven approach may lead to the development of tailored EV therapies, increasing therapeutic efficacy and avoiding off-target repercussions by means of personalized EV treatments catered to particular patient demands [153]. Moreover, extensive preclinical and clinical studies must thoroughly evaluate the long-term safety and effectiveness of EV-based therapy. EVs' probable immunogenicity, toxicity, and long-term effects must be thoroughly investigated to ensure their safe usage in clinical settings. From the laboratory to clinical practice, the advancement of EV-based therapeutics will rely mostly on regulatory issues, including guidelines for EV creation, characterization, and clinical use.

## 8 Conclusion

Given the uneven pathophysiology of IBD, which contrasts with the more homogeneous and accessible UC, the use of EVs in CD remains underinvested. This could be due to the unique patchy distribution of the disease in the intestine, making it more difficult to target specific areas compared to UC, which has a more uniform and accessible pathology. For CD, however, the growing field of EV research presents interesting therapy possibilities. EVs have shown remarkable immunomodulating properties, capacity to induce tissue regeneration, and a role in reducing colonic fibrosis—possibly key players in managing IBD. Though preclinical research and mouse models show promising outcomes, there are significant challenges in bringing EV-based treatments into clinical use. Among these include ensuring perfectly targeted delivery, standardizing isolation and characterization methods, optimizing scalable production processes, and managing difficult legal contexts. Overcoming these obstacles completely depends on strong validation derived from well-organized clinical investigations. Moreover, adding machine learning into these processes helps to optimize EV separation and increase targeted applications, thus improving therapeutic development. Dealing with these challenges will enable multidisciplinary cooperation to unlock the whole therapeutic potential of EVs for CD and other forms of IBD.

## CRediT authorship contribution statement

**George Chigozie Njoku:** Writing – review & editing, Writing – original draft, Visualization, Conceptualization. **Cathal Patrick Forkan:** Writing – review & editing, Writing – original draft. **Fumie Mitani Soltysik:** Writing – review & editing, Writing – original draft. **Peter Lindberg Nejsum:** Writing – review & editing, Writing – original draft. **Flemming Pociot:** Writing – review & editing, Writing – original draft, Funding acquisition, Conceptualization. **Reza Yarani:** Writing – review & editing, Writing – original draft, Visualization, Supervision, Project administration, Investigation, Funding acquisition, Conceptualization.

## Ethics approval and consent to participate

This review article does not require any ethical approval or allied consents for publication.

## Declaration of competing interest

The authors have no conflicts of interest to disclose and have all approved this submission.
